# The Mollusc Collection (Class Polyplacophora) at the Museum of Zoology, University of Concepción: curation status and value for integrative research

**DOI:** 10.3897/BDJ.14.e161845

**Published:** 2026-04-14

**Authors:** Jeremías Benjamín Norambuena Molina, Laura Tavera Martínez

**Affiliations:** 1 Museo de Zoología, Departamento de Zoología, Facultad de Ciencias Naturales y Oceanográficas, Universidad de Concepción, Casilla 160- C, Concepción, Chile Museo de Zoología, Departamento de Zoología, Facultad de Ciencias Naturales y Oceanográficas, Universidad de Concepción, Casilla 160- C Concepción Chile; 2 Postdoctoral Research, Departamento de Zoología, Facultad de Ciencias Naturales y Oceanográficas, Universidad de Concepción, Concepción, Chile Postdoctoral Research, Departamento de Zoología, Facultad de Ciencias Naturales y Oceanográficas, Universidad de Concepción Concepción Chile

**Keywords:** biological collections, Mollusca, Darwin Core, biodiversity, south-eastern Pacific, chitons, health index

## Abstract

**Background:**

Within a museum of biological collections, the care and periodic updating of collections represent a fundamental pillar of curatorial work. Keeping collections in good condition ensures better access for researchers interested in consulting or studying a specific taxonomic group. At the Museum of Zoology of the University of Concepción, a total of 140 samples belonging to the Polyplacophora class collection were curated. The first digitisation of this collection was carried out under the Darwin Core (DwC) standard, providing full accessibility to the respective collection data available in the GBIF and OBIS biodiversity databases. The collection consists of specimens collected between 1954 and 2022 in a wide range of localities, mainly in Chile and other countries such as Argentina, Ecuador and Norway, covering a total of 19 species. Additionally, in the year 2023, an evaluation of the collection’s health status was conducted, revealing that 42.857% of the samples had health levels of 5 or lower, while 57.143% were at level 6. By 2024, 96.429% of the collection improved to a health level of 7, while 3.571% remained at a health level of less than 5. These results indicate that the collection is in ideal conditions, detecting an improvement of the physical and digital material of the mollusc collection. This improvement enables researchers to more easily access information from the validated collection, supporting research in various areas such as conservation, resource management, climate change and biogeography.

**New information:**

This study represents the first report evaluating the health index of biological collections in Chile, showing how the prioritisation of actions requiring attention is important for the preservation of natural heritage. It also contributes to the current knowledge of biological collection databases in Chile, promoting the use of digital databases for better management of records and their information, so they can be used in future studies in various fields of natural sciences that require specific data.

## Introduction

**General value of biological collections and digitisation**

Biological collections represent invaluable repositories of past and recent biodiversity, preserving of their temporal and spatial records. This has enabled the use of this evidence in various fields in science (e.g. evolution, ecology, physiology, conservation and public health), contributing to the development of integrative research (Darrigran 2012). Currently, the perception of biological collections has gained greater relevance due to the increased digitisation of extended specimen data, such as photographs, tissue samples and associated biogeographic and ecological information. This process keeps collections updated and freely accessible for research without requiring a physical visit to the collection (Ortiz et al. 2023). Consequently, the concept of the "*extended specimen*" has been established, referring to all additional and related information about a specimen at the time of its collection. This includes tissue samples for DNA extraction, as well as morphological or ecological data for use in various research fields (Lendemer et al. 2019).

**The MZUC role and the focus on Class Polyplacophora**

In Chile, the Museum of Zoology of the University of Concepción (MZUC-CCC) contains one of the largest zoological collections in the country, consisting of 20 collections and approximately 800,000 specimens or samples. It serves as a significant biodiversity repository for students and researchers and is open to the entire community. Since 2016, the Museum of Zoology has been digitising its collections following the Darwin Core (DwC) biodiversity standard. This effort has resulted in the publication of 12 datasets in global biodiversity repositories, such as the Global Biodiversity Information Facility (GBIF) and the Ocean Biodiversity Information System (OBIS) (www.gbif.org/publisher/83685206-0bee-407b-8ed1-ac70087acf3f), increasing open access to databases for research and development across different scientific disciplines. Currently, the Mollusc Collection at the Museum of Zoology of the University of Concepción (UCCC_MZUC_MOL) represents the most significant marine collection, with a total of 30,000 individuals from various taxonomic groups and 395 type specimens, including holotypes, paratypes and topotypes (Artigas and Solar 2015). Since 2023, a continuous effort in curatorial work, updates and digitisation of the Mollusc Collection has been underway, starting with one of the most ancestral taxonomic groups within this phylum: the class Polyplacophora. This group of molluscs in the collection consists of samples donated by José Stuardo in 1970 and 2001, while more recent samples were donated by C. Aldea and J. R. Cárdenas in 2004 and by C. Cuevas in 2022. From the beginning of the collection in 1970 until 2023, the health status of the collection was unknown, considering the preservation methods and the quality of the recorded data.

**Health Index importance in biological collections**

The Health Index for biological collections, proposed by McGinley (1993), is a key tool for evaluating and monitoring the preservation and integrity of specimens over time. This index assesses physical integrity, storage conditions, documentation and sample accessibility, identifying issues, such as damage, chemical deterioration or documentation deficiencies. The detection of these problems enables the implementation of measures to ensure the long-term preservation of biodiversity and the integrity of the collections (Castaño-Ramírez and Ramírez-Chaves 2018). This leads to maintaining a robust Health Index, essential for the successful development of scientific research, as well-preserved collections in optimal conditions are fundamental for taxonomic, ecological and evolutionary studies. In this way, they provide fundamental historical data to understand the temporal and spatial changes in biodiversity (Garzón et al. 2022).

**Aim of the sudy**

During the first semester of 2024, the Mollusc Collection was reviewed again, with a significant effort made to improve the quality of digitised data and estimate the overall health status of the collection. Building on this effort, this study aims to characterise the Polyplacophora specimens in the Mollusc Collection of the Museum of Zoology at the University of Concepción, assessing their current condition and contributing to the understanding of Chile’s natural heritage and zoological reference collections. The digitised database, formatted according to DwC and published in GBIF and OBIS, is intended to serve as a publicly accessible information resource. It can be used as a reference resource of knowledge for research in various fields, such as ecology, biogeography, taxonomy and climate change.

## General description

### Purpose

**General information of the Polyplacophora Collection**

The Mollusc Collection, specifically the Polyplacophora group, is preserved both in wet conditions with 70% ethyl alcohol and in dry conditions, stored in wooden trays inside full-space metal cabinets. In total, the Polyplacophora group of the Mollusc Collection at the Museum of Zoology of the University of Concepción is represented by 140 samples containing 587 individuals distributed across 19 species. Amongst them, 16 species are found in Chile, while three species are represented by three samples and 20 individuals. Specimen sampling was carried out manually and/or through diving by various researchers, such as T. Cekalovic, J. Stuardo, C. Aldea and H. Moyano, who collected samples from depth ranges between 0 and 45 m. These specimens also originate from oceanic zones, bays, gulfs, ports and archipelagoes, including Chiloé Island, Tierra del Fuego and the Galápagos Islands.

**Specimen origin**

The samples that make up this collection have been collected from a total of five countries, primarily in Chile across 11 regions, including the Biobío Region, Los Lagos Region and Atacama Region. Notable collection sites in these areas include Lirquén,Talcán Island and Cocholgüe, representing the highest number of records with fifteen, nine and six entries, respectively. Additionally, four Polyplacophora samples originate from countries, such as Argentina, Ecuador and Norway. The oldest recorded specimen with the most precise sampling locality corresponds to a *Plaxiphora aurata* sample dating back to 1954, collected from Bulnes Fort in the Magallanes and Chilean Antarctic Region by T. Cekalovic. Since then, specimens have been added to the collection up until 2022, when the latest specimens were collected in Chile by M. Fuentes in Colcura (Biobío Region) and by J.R. Cárdenas and C. Aldea in the Gulf of Ancud (Los Lagos Region).

**Digitised data**

The digitised records of these specimens, formatted according to the Darwin Core (DwC) standard, include information like locality, preservation status and taxonomic identification. The digitised information includes the original taxonomic determinations made by researchers T. Cekalovic, J. Stuardo, C. Aldea, C. Valdovinos and H. Moyano, along with the taxonomic validation of each sample using the “Match Taxa” tool from the World Register of Marine Species (WoRMS) platform (Ahyong et al. 2024). The purpose of this validation was to update taxonomic groups and correct misidentifications in the collection. Subsequently, the data structure and quality in DwC format were validated using the OBIS and GBIF platforms (www.gbif.org/es/tools/data-validator), ensuring compliance with the Darwin Core standards (GBIF.org 2024). Furthermore, since most specimens lack coordinates or have incomplete ones, approximate decimal coordinates were assigned, based on sampling locality using the Google Earth Pro software.

## Geographic coverage

### Description

**Figure 1.**
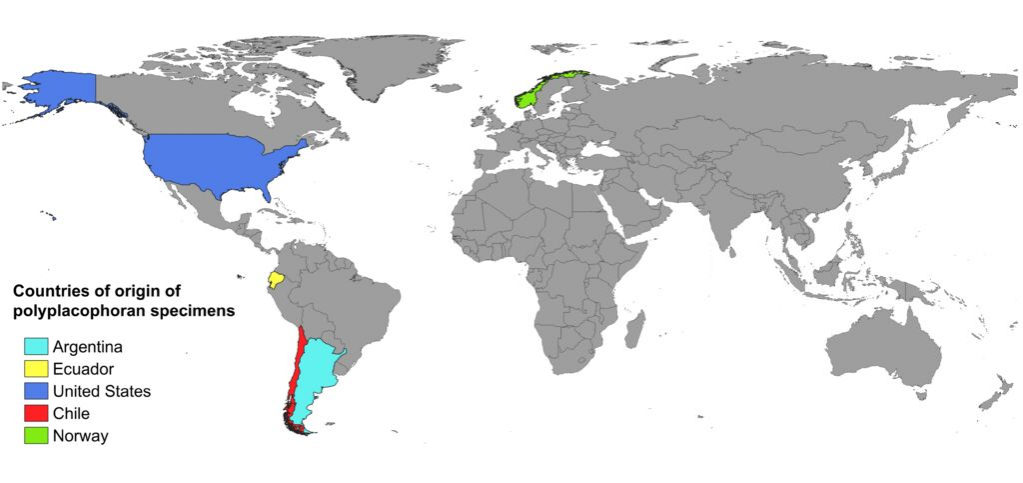
Geographical distribution of polyplacophoran specimens from the Mollusc Collection of the Museum of Zoology at the University of Concepción (MZUC-UCCC).

The geographic information is fully georeferenced in decimal degrees. Originally, most of the collection did not have georeferencing, with only 12 samples recorded with coordinates in degrees, minutes and seconds (DMS). Therefore, approximate coordinates were estimated, based on the sampling locality and converted to decimal format using the coordinate conversion tool from the Canadensys website (https://data.canadensys.net/tools/coordinates). The specimens were collected from various locations in different countries, primarily in Chile, with the most frequently recorded localities being Lirquén,Talcán Island, Cocholgüe, Pan de Azúcar and Guanaqueros (Table 1). Meanwhile, specimens obtained outside of Chile came from countries such as Argentina, Ecuador, Norway and the United States (Fig. 1).

### Coordinates

-55.08194 and 63.61835 Latitude; -109.45267 and 73.51022 Longitude.

## Taxonomic coverage

### Description

**Figure 2.**
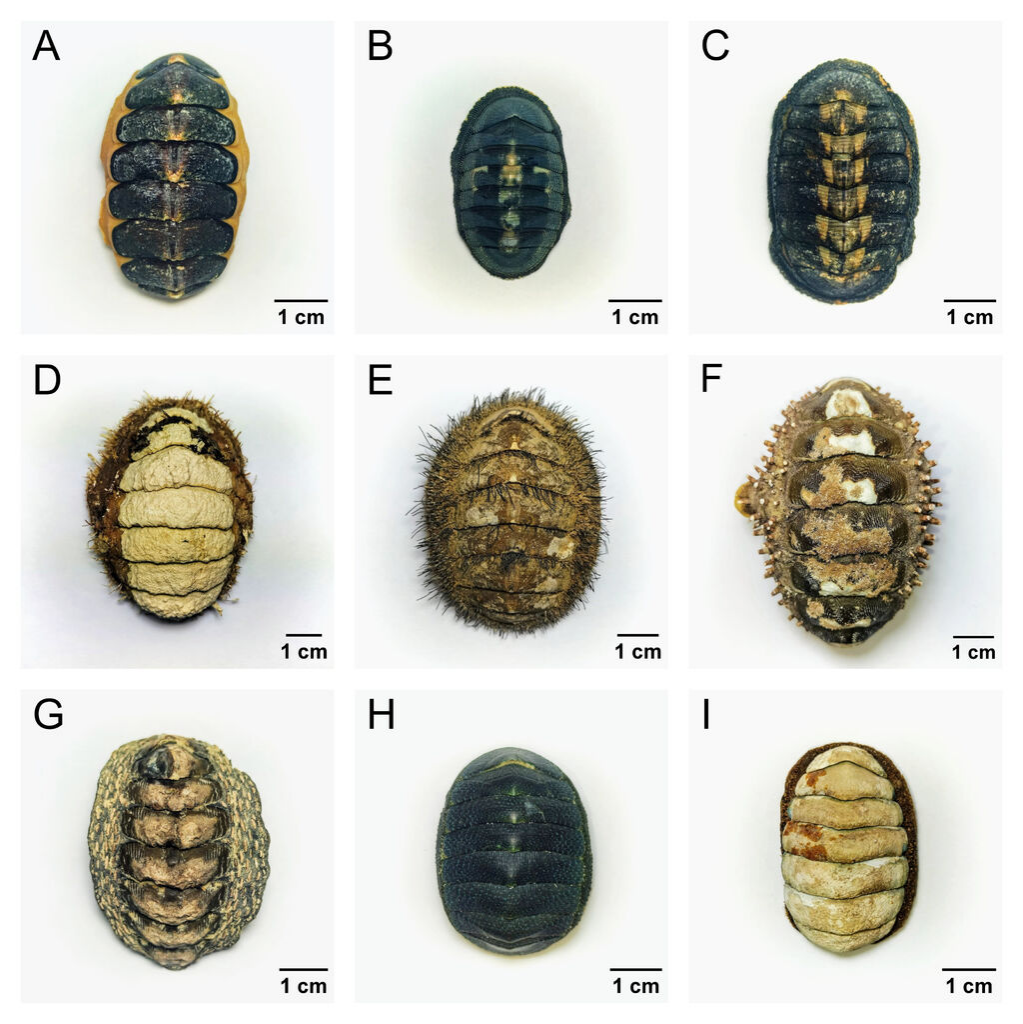
Representative species of Polyplacophora belonging to the Mollusc Collection of the Museum of Zoology of University of Concepción. **A** *Tonicia chilensis* (MZUC-UCCC 29044); **B** *Chiton cumingsii* (MZUC-UCCC 8207); **C** *Chiton granosus* (MZUC-UCCC 29039); **D** *Plaxiphora aurata* (MZUC-UCCC 10884); **E** *Chaetopleura peruviana* (MZUC-UCCC 19967); **F** *Enoplochiton echinatus* (MZUC-UCCC 8210); **G** *Enoplochiton niger* (MZUC-UCCC 19974); **H** *Chiton magnificus* (MZUC-UCCC 8209); **I** *Plaxiphora mercatoris* (MZUC-UCCC 15668). Photos taken by Jeremías Norambuena.

**Figure 3.**
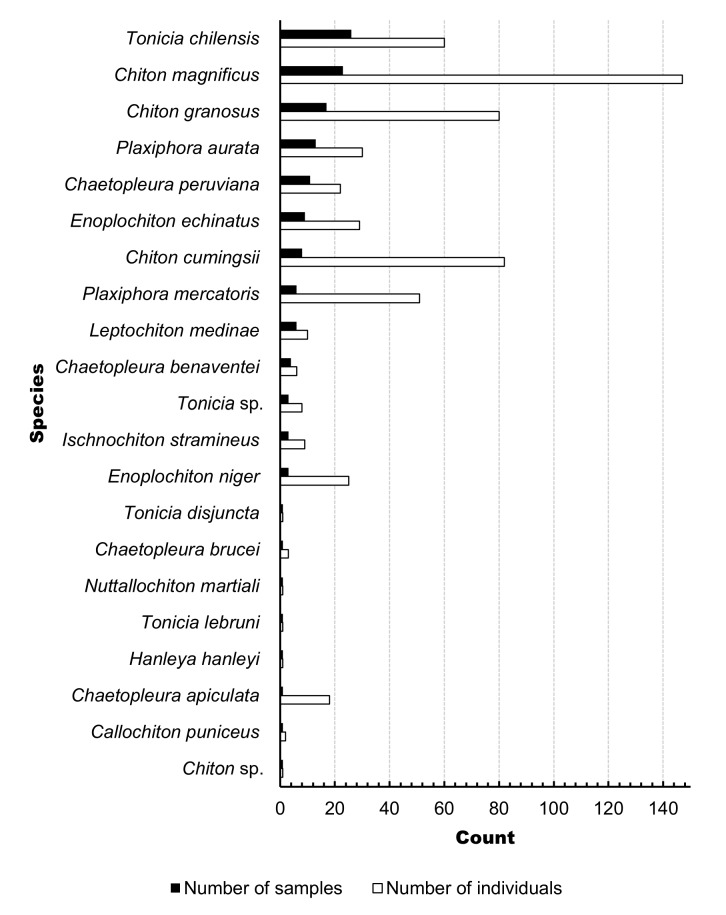
Number of samples and individuals per species of Polyplacophora from the Mollusc Collection of the Zoology Museum of the University of Concepción (MZUC-UCCC).

The Mollusc Collection of the class Polyplacophora comprises a total of 140 samples representing 19 species, distributed across three orders, seven families and 10 genera. Of these, 136 samples have been identified to the species level, while four have been identified only to the genus level. In this collection, the families Chitonidae and Chaetopleuridae stand out, with three of the most abundant genera represented by *Tonicia*, *Chiton* and *Chaetopleura*. The species *Tonicia chilensis*, *Chiton magnificus* and *Chiton granosus* are the most representative of the collection (Fig. 2). Importantly, the number of individuals per species does not always match the number of samples. For example, *Chiton cumingsii*, *Plaxiphora mercatoris* and *Plaxiphora aurata* have the highest number of individuals despite not being the most abundant in terms of sample count (Fig. 3).

### Taxa included

**Table:** 

Rank	Scientific Name	
species	*Callochiton puniceus*	
species	*Chaetopleura benaventei*	
species	*Chaetopleura brucei*	
species	*Chaetopleura apiculata*	
species	*Chaetopleura peruviana*	
species	*Chiton cumingsii*	
species	*Chiton granosus*	
species	*Chiton magnificus*	
species	*Enoplochiton echinatus*	
species	*Enoplochiton niger*	
species	*Tonicia disjuncta*	
species	*Tonicia lebruni*	
genus	* Tonicia *	
species	*Ischnochiton stramineus*	
species	*Nutallochiton martiali*	
species	*Plaxiphora aurata*	
species	*Plaxiphora mercatoris*	
species	*Leptochiton medinae*	
species	*Hanleya hanleyi*	
genus	* Chiton *	

## Temporal coverage

**Formation period:** 1954.

### Notes

**Figure 4.**
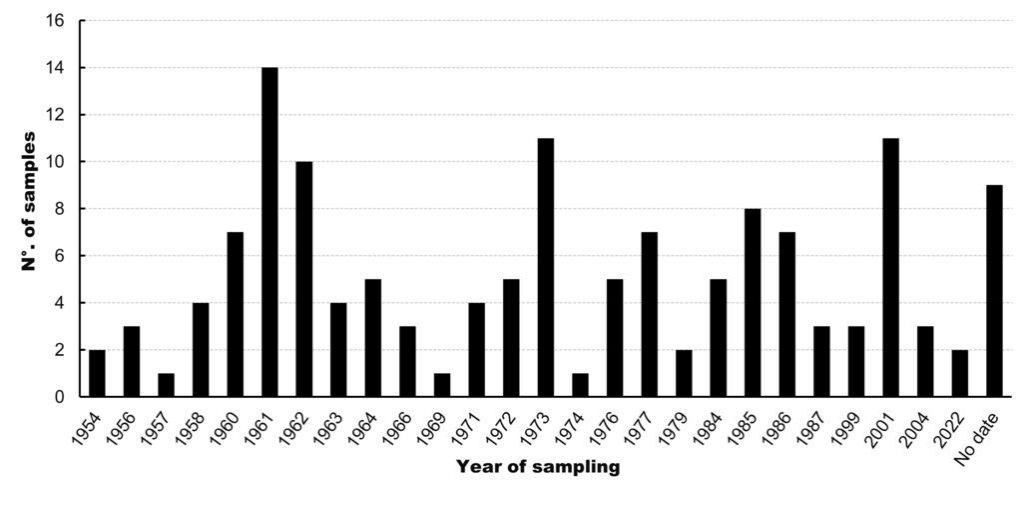
Number of polyplacophoran samples from the Mollusc Collection of the Museum of Zoology at the University of Concepción (MZUC-UCCC) collected in each sampling year.

The collection has a temporal range of collected specimens that begins in 1954 and are represented by a specimen of *P. aurata* obtained in the locality of Fuerte Bulnes in the Region of Magallanes and Chilean Antarctica, until the year 2022, when specimens of *L. medinae* and *C. granosus* were collected from the Gulf of Ancud and the locality of Colcura in the Los Lagos and Biobío Regions, respectively. The years with the highest number of records are 1961, with 14 samples, followed by 1973 and 2001, with 11 samples each (Fig. 4). The increase in the number of samples collected during these years was due to research cruises conducted in Chile by J. Stuardo on Isla Talcán in 1961, field trips from the Biology programme of the University of Concepción in Lirquén in 1973 and the sampling by C. Aldea, who collected specimens in the Tomé area (Biobío Region).

## Usage licence

### Usage licence

Creative Commons Public Domain Waiver (CC-Zero)

## Data resources

### Data package title

Colección de clase Polyplacophora del Museo de Zoología de la Universidad de Concepción UCCC_MZUC_MOL

### Resource link

https://doi.org/10.15468/qcndhj

### Alternative identifiers

https://ipt.iobis.org/esp-obis/resource?r=polyplacophora

### Number of data sets

1

### Data set 1.

#### Data set name

Colección de moluscos (clase Polyplacophora) del Museo de Zoología de la Universidad de Concepción

#### Data format

Darwin Core

#### Character set

140

**Data set 1.** 

Column label	Column description
occurrenceID	Unique sequential identifier code for each specimen.
basisOfRecord	“PreservedSpecimen” for all specimens.
institutionCode	"Museum of Zoology of the University of Concepción (UCCC_MZUC)" for all specimens.
collectionCode	“UCCC_MZUC_MOL” for all specimens.
catalogNumber	Unique number assigned to the specimen.
type	"PhysicalObject" for all specimens.
language	Spanish.
licence	CC BY-NC 4.0.
rightsHolder	Museum of Zoology of the University of Concepción.
institutionID	http://biocol.org/institution/museo-de-zoologia-universidad-de-Concepción.
collectionID	https://www.gbif.org/grscicoll/institution/c5ff6efa-e506-4126-8e21-cae8bc347041.
datasetName	Collection of Molluscs (Class Polyplacophora) of the Museum of Zoology of the University of Concepción.
recordedBy	Collector's name.
individualCount	Number of individuals assigned to the identification code.
occurrenceStatus	"Present" for all specimens.
preparations	Specimen preservation technique (In alcohol (ETOH) / Dry).
previousIdentifications	Previous identifications of the specimen.
samplingProtocol	Sampling protocol of the specimen.
eventDate	Full date (dd/mm/yy) of collection.
year	Full year where the event occurred.
month	Full month where the event occurred.
day	Full day where the event occurred.
eventRemarks	Collector's observations/comments associated with the record event.
continent	Continent where the specimen was collected.
waterBody	Waterbody where the specimen was collected.
islandGroup	Island group/Archipelago where the specimen was collected.
island	Island where the specimen was collected.
verbatimLocality	Name of the original locality written by the collector.
locationRemarks	Collector's observations/comments associated with the collection locality.
habitat	Environmental setting where the specimen was found during the collection event.
country	Country of origin where the specimen was collected.
countryCode	Country code of the collection location.
stateProvince	State; region; province or department where the specimen was collected.
county	Province; county or municipality where the specimen was collected.
municipality	Municipality or district where the specimen was collected.
locality	Common name used by the surrounding population to refer to the geographic area where the specimen was collected.
verbatimDepth	Original depth level written by the collector.
verbatimLatitude	Original latitude determined by the collector.
verbatimLongitude	Original longitude determined by the collector.
verbatimCoordinateSystem	"Degrees minutes seconds" for all specimens.
verbatimSRS	“WGS84” for all specimens.
decimalLatitude	Decimal latitude of the specimen converted to decimal coordinates.
decimalLongitude	Decimal longitude of the specimen converted to decimal coordinates.
geodeticDatum	“WGS84” for all specimens.
identifiedBy	Name(s) of the specimen's identifier(s).
dateIdentified	Date the specimen was identified.
scientificName	Species name of the specimen.
scientificNameID	Unique identifier code assigned by biodiversity databases (WoRMS) associated with the scientific name of the specimen.
acceptedNameUsage	Valid and currently used scientific name of the specimen.
kingdom	Scientific name of the kingdom to which the specimen belongs.
phylum	Scientific name of the phylum to which the specimen belongs.
class	Scientific name of the class to which the specimen belongs.
order	Scientific name of the order to which the specimen belongs.
family	Scientific name of the family to which the specimen belongs.
genus	Scientific name of the genus to which the specimen belongs.
specificEpithet	Suffix of the scientific name of the specimen.
taxonRank	Lowest taxonomic level determined for the specimen.
scientificNameAuthorship	Name and date of the describer who assigned the scientific name to the specimen.
vernacularName	Generic name by which the specimen is known.
minimumDepthInMetres	Minimum depth distance where the specimen was collected.
maximumDepthInMetres	Maximum depth distance where the specimen was collected.
coordinateUncertaintyInMetres	The horizontal distance in metres from the given coordinates describing the smallest circle containing the whole of the location.
georeferencedBy	A list of names of people, groups or organisations who determined the georeference for the location.
georeferencedDate	The date when the georeference was created.
georeferenceSources	A list of maps, gazetteers or other resources used to georeference the location.
georeferenceRemarks	Notes or comments about the spatial description determination, explaining assumptions made in addition or opposition to the those formalised in the method referred to the georeference.

## Additional information

### Collection Health Index (HI)

The evaluation of the collection's health status was conducted through a general review of the individuals contained in the Polyplacophora samples, using evaluation criteria based on the proposal by McGinley (1993) (Table 2). After evaluating each individual per sample, the results were averaged to obtain the overall health level of the collection.

**Initial status of Health Index in the Polyplacophora collection**

**Figure 5.**
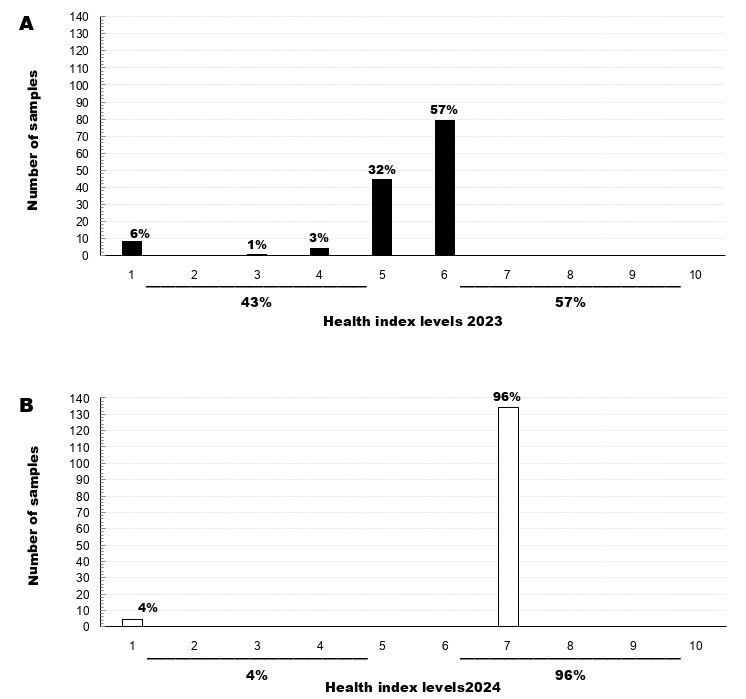
Health indices for the years 2023 and 2024 of the Polyplacophoran Mollusc Collection at the Museum of Zoology of the University of Concepción (MZUC-UCCC).

In 2023, this collection presented an average HI of 5.26, as, prior to the curation process, the material was identified and curated according to the museum's standards, with a total of 43% of the collection having a health level below 5 and 57% above level 5. A total of 45 samples were evaluated at level 5 and 80 at level 6 (Fig. 5). However, part of the collection was not curated properly, showing errors in taxonomic determination, maintenance or sample entry. As a result, nine samples were assigned level 1, one sample at level 3 and five samples at level 4.

**Re-assesment of Health Index and improvements**

After the curation process digitization of the samples and publication of the dataset in the GBIF and OBIS repositories in 2024, the HI was evaluated again, obtaining an average health level of 6.78 for the collection. A total of 135 samples raised their health level to level 7 equivalent to 96% of the total collection, except for 4%, corresponding to five samples evaluated at level 1 due to physical deterioration or lack of collection data, even though they are digitised (Fig. 5). These changes in the health levels of the collection between 2023 and 2024 were due to several updates and improvements, including taxonomic updates on labels and storage boxes, review of alcohol levels, restoration of damaged samples and reorganisation of dry samples on storage shelves. In addition, this was due to the entry into the collection of material not recorded in the museum's physical inventories (logbook and cards) and the digitisation of specimens in standard biodiversity formats (Darwin Core) (Norambuena Molina 2023).

**Health Index compared with other collections**

When comparing the HI reported in other collections, values have been recorded in some mammal collections with levels between 6 and 10 (Rivera-León et al. 2018, Castaño-Ramírez and Ramírez-Chaves 2018, Leuro-Robles et al. 2020), fish collecions with an HI of 8 (Henao-Osorio et al. 2022), amphibian collections with a value of 7 (Cárdenas and Delgadillo 2019) and insect collections with levels of 6 (Ward and Malysheva 2022), all considered acceptable according to the Moser et al. (2000) scale in the collections of the National Museum of Natural History of the United States (Favret 2007). Therefore, it follows that the collection of Polyplacophora molluscs records a very similar HI to most of the reported collections. This highlights the total lack of evaluation of health indices in marine invertebrate collections, making it difficult to compare the HI with other mollusc collections. Nevertheless, the HI of the polyplacophoran mollusc collection, which grouped a greater proportion of samples in 2024 (96% at level 7), indicates that the collection is in ideal conditions, as 70% or more of the collection should register levels above 6, according to Simmons and Muñoz-Saba (2005). Therefore, in this case, the absence of samples at levels 8-10 demonstrates that improvement measures for this collection are more closely related to the use of digitised information for the development of research in taxonomy or biogeography and publications that include type material. Currently, no publications have specifically described the health index in mollusc collections. However, the publication by Favret (2007) evaluates the health index of several collections, including one of molluscs, where it studies the quality levels in aspects of specimen conservation, such as storage conditions, data quality and digitisation status, concluding that the HI levels are acceptable.

### Contributions of the Mollusc Collection - Class Polyplacophora from MZUC-UCCC

This resource constitutes an important natural heritage of marine biodiversity, not only from Chile, but also from other locations worldwide. It is part of an important repository that preserves reference material identified and donated by renowned researchers, such as T. Cekalovic, J. Stuardo, C. Aldea and H. Moyano. From 2023 to the present, the Mollusc Collection continues to grow and to advance in its maintenance, conservation and digitisation in standard formats, together with other taxonomic groups of molluscs. Therefore, this dataset corresponds to the first publication in OBIS and GBIF from a Mollusc collection in Chile, promoting the development of open-access marine biodiversity databases for the south-eastern Pacific, with validated information available for use in studies focused on ecology, biogeography and conservation. On the other hand, the evaluation of the current state of the polyplacophore mollusc collection through a quantitative index corresponds to the first report of this index for biological collections in Chile. This provides valuable information, making it possible to evaluate and compare the progress of biological collections over time and to guide efforts to ensure their preservation.

### Conclusions

Through this work, all available information from the Mollusc (Class Polyplacophora) Collection of the Museum of Zoology at the University of Concepción was compiled, thus generating an important source of knowledge about the Phylum Mollusca, not only at national, but also at international level. The state of health of the biological collections in Chile allows prioritising the actions that require greater attention to ensure the optimal preservation of the natural heritage. It also promotes the use of databases of biological collection for the development of integrative research. This serves as a robust source of primary information for studies in diverse areas of the natural sciences and bioresource management, acting as a tool for decision-makers at the political level.

## Figures and Tables

**Table 1.** Most representative sampling localities of polyplacophorans from the Mollusc Collection of the Museum of Zoology at the University of Concepción.

**Country**	**Locality**	**Number of samples**
Chile	Lirquén	15
	Talcán Island	9
	Cocholgüe	6
	Pan de Azúcar	5
	Isla Guarello	4
	Pumal**í**n	4
	Tumbes Peninsula	4
	Rocoto Beach	3
	Chope Estuary	3
	Guanaqueros	3
Argentina	Deseado port	1
Noruega	Trondheim Fjord	1
Ecuador	Galapagos Islands	1
United States	Beaufort	1

**Table 2.** Levels and evaluation criteria for the health index.

**Health Level**	**Description**
**1**	Deteriorated specimens, curable or low priority.
**2**	Prepared specimens, but not consultable.
**3**	Unidentified material, but accessible.
**4**	Identified material, but not entered.
**5**	Identified specimens, but with incomplete curation.
**6**	Identified and properly curated material.
**7**	Specimens inventoried at the species level, including physical and digital records.
**8**	Samples with ecological and ethological data, used in taxonomic, biogeographical or natural history studies.
**9**	Material with information associated with publications.
**10**	Samples included in publications and catalogued as holotypes, allotypes and others, at level 7 or higher.
